# Supplementary figures and images for: Different renoprotective effects of luseogliflozin depend on the renal function at the baseline in patients with type 2 diabetes: A retrospective study during 12 months before and after initiation
Source: PLoS One. 2021 Mar 15;16(3):e0248577. doi: 10.1371/journal.pone.0248577 (PMC7959360; doi:10.1371/journal.pone.0248577)

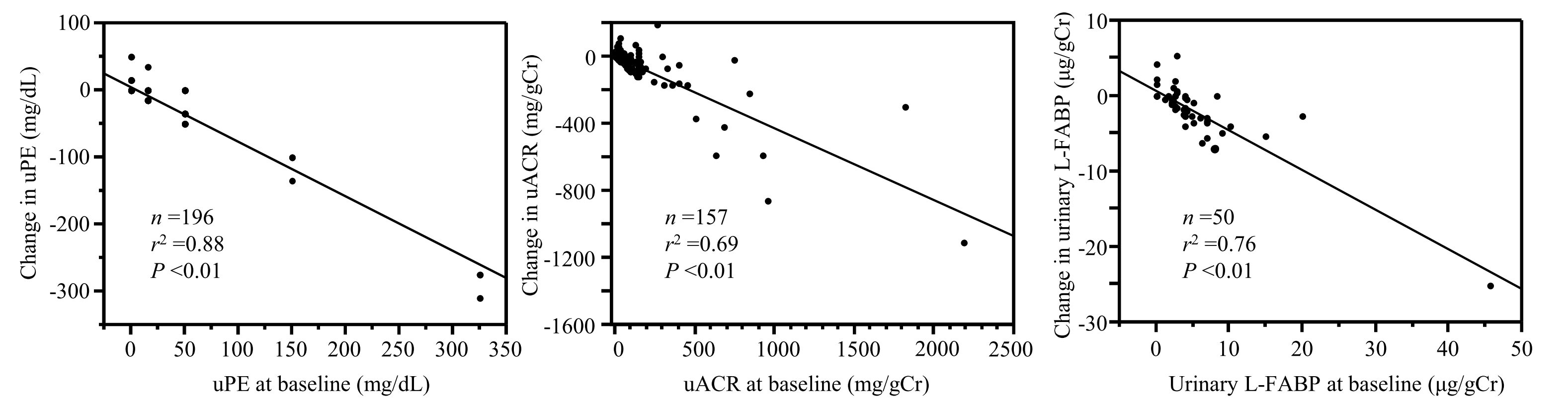

Supplement: S1 Fig — uPE, urinary protein excretion; uACR, urinary albumin-to-creatinine ratio; L-FABP, liver-type fatty acid-binding protein. (TIF) [file pone.0248577.s002.tif]
