# Supplementary material for: Different renoprotective effects of luseogliflozin depend on the renal function at the baseline in patients with type 2 diabetes: A retrospective study during 12 months before and after initiation
Source: PLoS One. 2021 Mar 15;16(3):e0248577. doi: 10.1371/journal.pone.0248577 (PMC7959360; doi:10.1371/journal.pone.0248577)
Supplement: S1 Table — (DOCX) [file pone.0248577.s004.docx]

**S1 Table:** Changes in the eGFR in 145 patients who have been treated over 12 months before the initiation of luseogliflozin administration.

| eGFR | All subjects | Groups according to eGFR at baseline | | |
| --- | --- | --- | --- | --- |
| (mL/min/1.73 m^2^) |  | High eGFR group | Normal eGFR group | Low eGFR group |
|  | (*n*=145) | (*n*=35) | (*n*=81) | (*n*=29) |
| -12 months | 78±20* | 102±16 | 76±11 | 53±12** |
| -9 months | 77±19* | 98±14 | 77±12* | 52±11** |
| -6 months | 77±20 | 98±14* | 76±12 | 52±11** |
| -3 months | 78±21* | 102±16 | 76±11* | 51±11** |
| Baseline (0 months) | 76±20 | 102±9 | 75±8 | 49±9 |
| 1 month | 74±20** | 96±13** | 74±11 | 48±9 |
| 3 months | 74±19** | 97±12** | 73±11* | 49±11 |
| 6 months | 74±20** | 97±13** | 73±11* | 49±11 |
| 9 months | 75±20 | 99±12* | 74±12 | 50±10 |
| 12 months | 75±19 | 97±11** | 74±13 | 50±11 |
| Differences from baseline |  |  |  |  |
| Before the initiation | -2±9 | -0±12 | -1±8 | -5±7 |
| After the initiation | -1±9 | -5±9 | -0±10# | +2±6## $$ |

eGFR, estimated glomerular filtration rate

* *P*<0.05, ** *P*<0.01 vs. corresponding value at baseline, # *P*<0.05, ## *P*<0.01 vs. corresponding value in the high eGFR group, $$ *P*<0.01 vs. corresponding value before the initiation
