# Supplementary material for: Different renoprotective effects of luseogliflozin depend on the renal function at the baseline in patients with type 2 diabetes: A retrospective study during 12 months before and after initiation
Source: PLoS One. 2021 Mar 15;16(3):e0248577. doi: 10.1371/journal.pone.0248577 (PMC7959360; doi:10.1371/journal.pone.0248577)
Supplement: S2 Table — (DOCX) [file pone.0248577.s005.docx]

**S2 Table:** Adverse events that caused discontinuation of luseogliflozin in the safety analysis set.

| No. | eGFR group | Period† (days) | Age/sex | Adverse event |
| --- | --- | --- | --- | --- |
| 1 | High eGFR | 4 | 23/male | Nausea |
| 2 | High eGFR | 15 | 51/female | Skin eruption |
| 3 | Normal eGFR | 21 | 71/female | Skin eruption |
| 4 | Normal eGFR | 31 | 51/male | Nausea, vomiting |
| 5 | High eGFR | 35 | 47/male | Pollakisuria |
| 6 | Low eGFR | 42 | 79/male | Eosinophilia |
| 7 | Low eGFR | 42 | 70/female | Dizziness |
| 8 | Normal eGFR | 49 | 70/female | Pollakisuria |
| 9 | High eGFR | 63 | 53/female | Skin ulcer |
| 10 | Normal eGFR | 63 | 53/male | Diarrhea |
| 11 | High eGFR | 83 | 50/male | Thirst |
| 12 | Normal eGFR | 90 | 65/male | Genital infection |
| 13 | Normal eGFR | 91 | 33/male | Nausea |
| 14 | Low eGFR | 91 | 69/female | Pollakisuria |
| 15 | Low eGFR | 94 | 70/male | Sepsis |
| 16 | Normal eGFR | 100 | 82/male | Dehydration |
| 17 | Normal eGFR | 114 | 65/male | Sudden death |
| 18 | Normal eGFR | 118 | 62/male | Bladder tumor |
| 19 | Low eGFR | 125 | 72/male | Pleural mesothelioma |
| 20 | High eGFR | 141 | 66/male | Prostatic cancer |
| 21 | Normal eGFR | 161 | 70/female | Pancreatic cancer |
| 22 | Low eGFR | 169 | 75/female | Uremia |
| 23 | High eGFR | 183 | 71/female | Pancreatic cancer |
| 24 | Low eGFR | 203 | 76/female | Kidney injury |

eGFR, estimated glomerular filtration rate

† Period indicates the administration period after the initiation of luseogliflozin.
