# Supplementary material for: Different renoprotective effects of luseogliflozin depend on the renal function at the baseline in patients with type 2 diabetes: A retrospective study during 12 months before and after initiation
Source: PLoS One. 2021 Mar 15;16(3):e0248577. doi: 10.1371/journal.pone.0248577 (PMC7959360; doi:10.1371/journal.pone.0248577)
Supplement: S3 Table — (DOCX) [file pone.0248577.s006.docx]

**S3 Table:** Adverse events during the observation period in the safety analysis set.

|  | All subjects | Groups according to eGFR at baseline | | | *P* |
| --- | --- | --- | --- | --- | --- |
|  |  | High eGFR | Normal eGFR | Low eGFR |  |
|  | (*n*=238) | (*n*=60) | (*n*=131) | (*n*=47) |  |
| Urogenital infection | 4 (2%) | 1 (2%) | 2 (2%) | 1 (2%) | 0.96 |
| Severe hypoglycemia | 0 | 0 | 0 | 0 | - |
| Increased urine volume | 11 (5%) | 3 (5%) | 4 (3%) | 4 (9%) | 0.31 |
| Volume depletion | 12 (5%) | 4 (7%) | 3 (2%) | 5 (11%) | 0.06 |
| Cerebral infarction | 3 (1%) | 0 | 1 (1%) | 2 (4%) | 0.11 |
| Skin itching/eruption | 9 (4%) | 3 (5%) | 2 (2%) | 4 (9%) | 0.08 |
| Gastrointestinal symptoms† | 12 (5%) | 3 (5%) | 6 (5%) | 3 (6%) | 0.89 |
| Neoplasm | 5 (2%) | 1 (2%) | 3 (2%) | 1 (2%) | 0.96 |
| Death | 2 (1%) | 0 | 1 (1%) | 1 (2%) | 0.48 |
| Others | 28 (12%) | 3 (5%) | 15 (11%) | 10 (21%) | 0.03 |
| Total | 86 (36%) | 18 (30%) | 37 (28%) | 31 (66%) | <0.01 |

eGFR, estimated glomerular filtration rate

† Gastrointestinal symptoms include nausea, vomiting, abdominal fullness, abdominal pain, constipation and diarrhea.
