# Supplementary material for: Different renoprotective effects of luseogliflozin depend on the renal function at the baseline in patients with type 2 diabetes: A retrospective study during 12 months before and after initiation
Source: PLoS One. 2021 Mar 15;16(3):e0248577. doi: 10.1371/journal.pone.0248577 (PMC7959360; doi:10.1371/journal.pone.0248577)
Supplement: Attachment — Submitted filename: Response to Reviewers.docx [file pone.0248577.s007.docx]

**Response to the academic editor**

*1. Please ensure that your manuscript meets PLOS ONE's style requirements, including those for file naming. The PLOS ONE style templates can be found at*

*https://journals.plos.org/plosone/s/file?id=wjVg/PLOSOne_formatting_sample_main_body.pdf and*

*https://journals.plos.org/plosone/s/file?id=ba62/PLOSOne_formatting_sample_title_authors_affiliations.pdf*

Response: We amended the manuscript to meet PLOS ONE’s style requirements.

*2. We note that your retrospective study was approved in 2018 but that data was collected for patients in 2019. Please clarify whether the ethical approval included permission to keep gathering retrospective data after approval.*

Response: We apologize for the mistake in the year. We amended the description (2019 -> 2018) in the **Study design and patients** and **Figure 1** in the revised manuscript.

*3. Thank you for stating in your Funding Statement:*

*"This work was partly supported by Taisho Pharmaceutical Co., Ltd (Tokyo, Japan)."*

*Please provide an amended statement that declares *all* the funding or sources of support (whether external or internal to your organization) received during this study, as detailed online in our guide for authors at http://journals.plos.org/plosone/s/submit-now. Please also include the statement “There was no additional external funding received for this study.” in your updated Funding Statement.*

*Please include your amended Funding Statement within your cover letter. We will change the online submission form on your behalf.*

Response: We amended the Funding Statement in the revised manuscript and added the Funding Statement within our cover letter.

*4. We note that the grant information you provided in the ‘Funding Information’ and ‘Financial Disclosure’ sections do not match.*

*When you resubmit, please ensure that you provide the correct grant numbers for the awards you received for your study in the ‘Funding Information’ section.*

Response: We amended the Funding Information although the grant number was not issued.

*5. Thank you for stating the following in the Competing Interests section:*

*"H Ito has received lecture fees from Taisho Pharmaceutical Co., Ltd., Eli Lilly Japan KK, Boehringer Ingelheim, Takeda Pharmaceutical Company Ltd., Sanofi KK, Novo Nordisk Pharma Ltd., MSD KK, Novartis Pharma KK, Astellas Pharma, Daiichi Sankyo Company, Terumo Corporation, Mochida Pharmaceuticals, Teijin Pharma, Kissei Pharmaceuticals, Kowa Pharmaceuticals, Mitsubishi Tanabe Pharma Corporation, Sanwa Kagaku Kenkyusho, Dainippon Sumitomo Pharma, AstraZeneca KK, Kyowa Hakko Kirin, Shionogi and Co, Otsuka Pharmaceutical Co., Ltd., Bayer Yakuhin, Ltd., and Santen Pharmaceutical Co., Ltd., and has received consulting fee from Becton, Dickinson and Company. S Matsumoto has received lecture fees from Novo Nordisk Pharma Ltd., Astellas Pharma, and AstraZeneca KK. T Izutsu has received lecture fees from Sanofi KK, Taisho Pharmaceutical Co., Ltd., Kyowa Hakko Kirin, Bayer Yakuhin, Ltd., and Mitsubishi Tanabe Pharma Corporation. S Antoku has received lecture fees from Kyowa Hakko Kirin, Sanofi KK, Kyowa Hakko Kirin, Taisho Pharmaceutical Co., Ltd., Daiichi Sankyo Company, and Otsuka Pharmaceutical Co., Ltd. E Kusano, J Kondo, H Inoue, T Yamasaki, T Mori and M Togane have no conflict of interest."*

*We note that you received funding from a commercial source: Taisho Pharmaceutical Co., Ltd.*

*Please provide an amended Competing Interests Statement that explicitly states this commercial funder, along with any other relevant declarations relating to employment, consultancy, patents, products in development, marketed products, etc.*

*Within this Competing Interests Statement, please confirm that this does not alter your adherence to all PLOS ONE policies on sharing data and materials by including the following statement: "This does not alter our adherence to PLOS ONE policies on sharing data and materials.” (as detailed online in our guide for authors http://journals.plos.org/plosone/s/competing-interests). If there are restrictions on sharing of data and/or materials, please state these. Please note that we cannot proceed with consideration of your article until this information has been declared.*

*Please include your amended Competing Interests Statement within your cover letter. We will change the online submission form on your behalf.*

*Please know it is PLOS ONE policy for corresponding authors to declare, on behalf of all authors, all potential competing interests for the purposes of transparency. PLOS defines a competing interest as anything that interferes with, or could reasonably be perceived as interfering with, the full and objective presentation, peer review, editorial decision-making, or publication of research or non-research articles submitted to one of the journals. Competing interests can be financial or non-financial, professional, or personal. Competing interests can arise in relationship to an organization or another person. Please follow this link to our website for more details on competing interests: http://journals.plos.org/plosone/s/competing-interests*

Response: We amended the Competent interest Statement in the revised manuscript and added the Competent interest Statement within our cover letter.

**Response to the reviewer #1**

*1. One of the limitations was the removal of 36 patients from the study based on adverse events occurring in the first 100 days. These patients could have been included in an Intention-to-treat analysis. Also, the adverse events in this group were renal related as well which could have affected overall study data.*

Response: Because we cannot analyze the clinical data during 12 months in the subjects whose luseogliflozin administration was stopped, groups according to the baseline eGFR were added to **S5 Table** of the revised manuscript. Adverse events related to the renal prognosis, such as uremia and kidney injury were observed in the low eGFR group. Thus, we added an explanation to the **Discussion** section of the revised manuscript indicating that careful interpretation is required in relation to the renoprotective effect of luseogliflozin in the low eGFR group when considering subjects, including the SAS.

*2. The study population was skewed towards men, with 71% of the study population being men. Is it related to baseline demographics in the Japanese population?*

Response: In 2017, the prevalence of diabetes in the Japanese adult population was greater in men (men, 18.1%; women, 10.5%) (reference 10). However, the proportion of men in this study seems to be higher in comparison to our other studies, which were conducted around the same time (58% in reference 30,39) as the reviewer pointed out. Although there are no previous reports investigating sex differences in the prevalence of obesity in Japanese diabetic patients, obesity is predominant in all generations of the Japanese general population (reference 10). Because it is highly likely that obese subjects were selected for treatment using SGLT2 inhibitors, the difference in the prevalence of obesity between men and women appears to have caused a sex difference in the subjects of the present study

We added an explanation about these points to the **Discussion** section of the revised manuscript.

*3. More adverse effects occurred in the low eGFR group, which is important, even though the authors state that these were not necessarily SGLT-2 related*

Response: In the **Discussion** section of the revised manuscript, we added a passage noting that caution is required to detect adverse events in the low eGFR group, as the reviewer suggested.

*4. In most studies of SGLT-2 inhibitors, there is a transient decline in GFR, and this normalizes over time. What is your hypothesis for the persistent decline in the high and normal eGFR groups?*

Response: In this study, the eGFR showed a transient decline after the start of luseogliflozin, and did not decrease thereafter in the high eGFR group or recovered to the baseline value in the normal eGFR group (**S4 Table**). We added a passage indicating that these changes did not contradict the previous studies to the **Discussion** section of the revised manuscript.

*5. There are grammatical and typographical errors that will need to be fixed. Minor changes.*

Response: The manuscript was revised after editing by a professional editor who is a native speaker of English.

**Response to the reviewer #2**

*Line 83- add "with" in front of diabetes*

Response: We added “with” in front of diabetes in the revised manuscript.

*Line 215- Replace "Whole" with "All" and in other parts of manuscript if other reviewers and editors also agree*

Response: We replaced “whole” to “all” in the revised manuscript.
